# Facilitating GL13K Peptide Grafting on Polyetheretherketone via 1-Ethyl-3-(3-dimethylaminopropyl)carbodiimide: Surface Properties and Antibacterial Activity

**DOI:** 10.3390/ijms23010359

**Published:** 2021-12-29

**Authors:** Chih-Chien Hu, Selvaraj Rajesh Kumar, Truong Thi Tuong Vi, Yu-Tzu Huang, Dave W. Chen, Shingjiang Jessie Lue

**Affiliations:** 1 Division of Join Reconstruction, Department of Orthopedics, Chang Gung Medical Center at Linkou, Guishan District, Taoyuan City 333, Taiwan; chihchienhu@hotmail.com; 2 Department of Chemical and Materials Engineering, Chang Gung University, Guishan District, Taoyuan City 333, Taiwan; rajeshkumarnst@gmail.com; 3 Division of Pediatric Gastroenterology and Hepatology, Department of Pediatrics, Chang Gung Memorial Hospital, Guishan District, Taoyuan City 333, Taiwan; truongthituongvi005@gmail.com; 4 Department of Chemical Engineering, Chung Yuan Christian University, Zhongli, Taoyuan City 320, Taiwan; yt_huang@cycu.edu.tw; 5 R&D Center for Membrane Technology and Research Center for Circular Economy, Chung Yuan Christian University, Zhongli, Taoyuan City 320, Taiwan; 6 Department of Orthopedic Surgery, Chang Gung Memorial Hospital, Keelung City 204, Taiwan; mr5181@cgmh.org.tw; 7 Department of Safety, Health and Environment Engineering, Ming Chi University of Technology, Taishan District, New Taipei City 243, Taiwan

**Keywords:** antimicrobial peptides, antibacterial activities, biofilm resistance, orthopedic implants

## Abstract

In the present work, the antimicrobial peptide (AMP) of GL13K was successfully coated onto a polyetheretherketone (PEEK) substrate to investigate its antibacterial activities against *Staphylococcus aureus* (*S. aureus*) bacteria. To improve the coating efficiency, 1-ethyl-3-(3-dimethylaminopropyl)carbodiimide (EDC) was mixed with a GL13K solution and coated on the PEEK surface for comparison. Both energy-dispersive X-ray spectroscopy (EDX) and X-ray photoelectron spectroscopy (XPS) data confirmed 30% greater peptide coating on PEEK/GL13K-EDC than PEEK without EDC treatment. The GL13K graft levels are depicted in the micrograms per square centimeter range. The PEEK/GL13K-EDC sample showed a smoother and lower roughness (Rq of 0.530 µm) than the PEEK/GL13K (0.634 µm) and PEEK (0.697 µm) samples. The surface of the PEEK/GL13K-EDC was more hydrophilic (with a water contact angle of 24°) than the PEEK/GL13K (40°) and pure PEEK (89°) samples. The pure PEEK disc did not exhibit any inhibition zone against *S. aureus*. After peptide coating, the samples demonstrated significant zones of inhibition: 28 mm and 25 mm for the PEEK/GL13K-EDC and PEEK/GL13K samples, respectively. The bacteria-challenged PEEK sample showed numerous bacteria clusters, whereas PEEK/GL13K contained a little bacteria and PEEK/GL13K-EDC had no bacterial attachment. The results confirm that the GL13K peptide coating was able to induce antibacterial and biofilm-inhibitory effects. To the best of our knowledge, this is the first report of successful GL13K peptide grafting on a PEEK substrate via EDC coupling. The present work illustrates a facile and promising coating technique for a polymeric surface to provide bactericidal activity and biofilm resistance to medical implantable devices.

## 1. Introduction

In recent years, biofilm-associated microbial infections on implantable medical devices have been a major concern and placed high burdens on healthcare systems. The implantable biomaterials include stainless steel, titanium (Ti), tantalum, zirconia, alumina, polyethylene, polyurethane, polytetrafluoroethylene (PTFE), and polyetherketoneketone (PEKK) [1,2,3,4]. Among these, the thermoplastic polyetheretherketone (PEEK) is a state-of-the-art material due to its superior chemical resistance, thermal stability, mechanical strength similar to cortical bone, and radiolucent property [5,6]. PEEK has the potential to replace traditional ceramic and metal- or alloy-based implantable devices for dental, spinal, joint, and orthopedic applications [7,8,9,10]. However, the hydrophobicity of the pure PEEK increases the chance of biofilm formations and may not eliminate post-surgical bacterial infection [11,12].

In addition to antibiotic administration [13], which involves the risk of developing antibiotic resistance, bio-inspired peptides with antimicrobial characteristics are a novel approach due to their wide range of activity against fungi, bacteria, and viruses. Antimicrobial peptides (AMPs) exhibit negligible toxicity to mammalian cells and low bacterial resistance [14]. AMPs are generally amphipathic and cationic and have an antimicrobial action when contacting bacterial cell membranes [15,16,17,18].

Among the many AMPs, GL13K is a potent peptide against dental- and bone-related pathogenic bacteria in a solid substrate [19] and in a solution medium [20]. In the solution phase, most of the antibacterial studies have focused on the minimum inhibition concentrations of GL13K [21,22,23] to estimate the quantitative information. To fabricate a porous polymer structure, the GL13K peptide was mixed into mineralized collagen scaffolds [24] or a pectin-coated chitosan nanofiber membrane [25]. In addition, this AMP was coated on titanium (Ti) substrates to investigate its antibacterial activities and biofilm resistance [19,26,27,28]. However, GL13K coating on a Ti surface is a complex process which involves polishing with SiC paper, etching with NaOH, or O_2_ plasma treatment to activate the surface for peptide physical loading or covalent bonding [14,19,28,29].

Other AMPs than GL13K were coated on PEEK substrates [17,30,31,32,33]. The conventional peptide coating procedure for a PEEK surface involves roughing using sandpaper, plasma spraying, and alkaline and sulfonating or acid treatments to activate the PEEK surface [7,34,35]. However, the usage of sandpaper induces scratches and pinhole cracks on the PEEK surface, whereas a plasma spray may alter the polymeric chains. The acid treatment also induces a porous structure that might possibly decrease its mechanical strength, have a corrosive effect, and change the PEEK chemical compositions [36]. To date, the direct grafting or coating of AMP on a PEEK surface is challenging and needs more investigation.

In this study, GL13K was directly coated onto a PEEK surface using the facile wet chemical method, which is especially suitable for substrates with irregular shapes. The GL13K was mixed with a 1-ethyl-3-(3-dimethylaminopropyl)carbodiimide (EDC) coupling agent for PEEK substrate immersion to induce conjugation. Samples without EDC coupling were prepared for comparison. The physicochemical, micrograph, and surface properties of the samples were assessed to validate the GL13K coating on the PEEK. The GL13K-coated PEEK samples were challenged with *S. aureus* bacteria to investigate the antibacterial effects and its biofilm resistance properties. To the best of our knowledge, this is the first report of successful GL13K peptide grafting on a PEEK substrate to investigate the antibacterial and biofilm resistance properties.

## 2. Results and Discussion

### 2.1. Morphological Analysis

**Figure 1:**
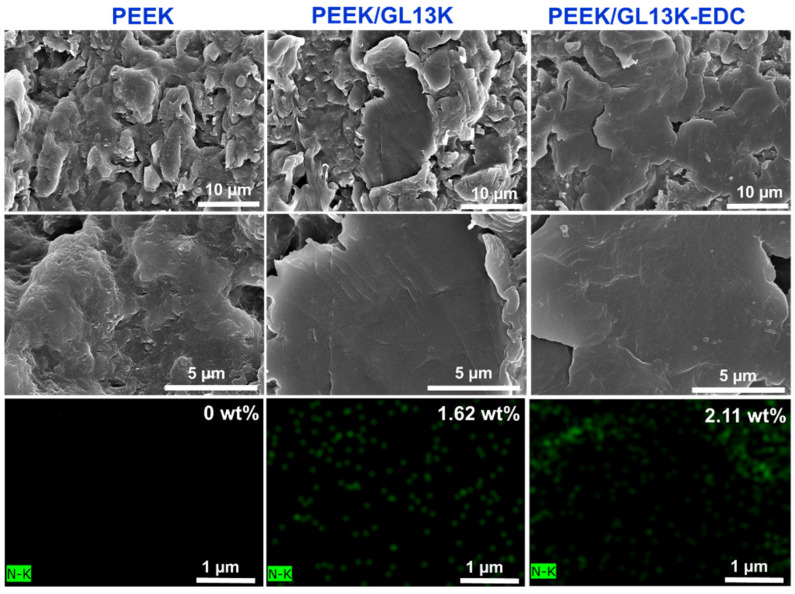
Field emission scanning electron microscopic (FESEM) images (top two rows) and nitrogen mapping from energy dispersive X-ray spectroscopy (EDX) micrographs (last row) of PEEK, PEEK/GL13K, and PEEK/GL13K-EDC samples.

Low- and high-magnification surface micrographs of the PEEK, PEEK/GL13K, and PEEK-EDC/GL13K samples were scrutinized using FESEM (Figure 1). The pure PEEK displayed a rough, bumpy surface. After coating the PEEK with GL13K, a smooth surface morphology was obtained. This confirmed the successful physical attachment of GL13K onto the PEEK surface. In the case of the PEEK/GL13K-EDC sample, a smoother surface was observed due to the homogeneous peptide coating on the entire sample area. Hence, more uniform peptide coating effects were observed following the chemical conjugation techniques through EDC coupling compared with the PEEK/GL13K sample (as was clearly visible at a low magnification).

**Figure 2:**
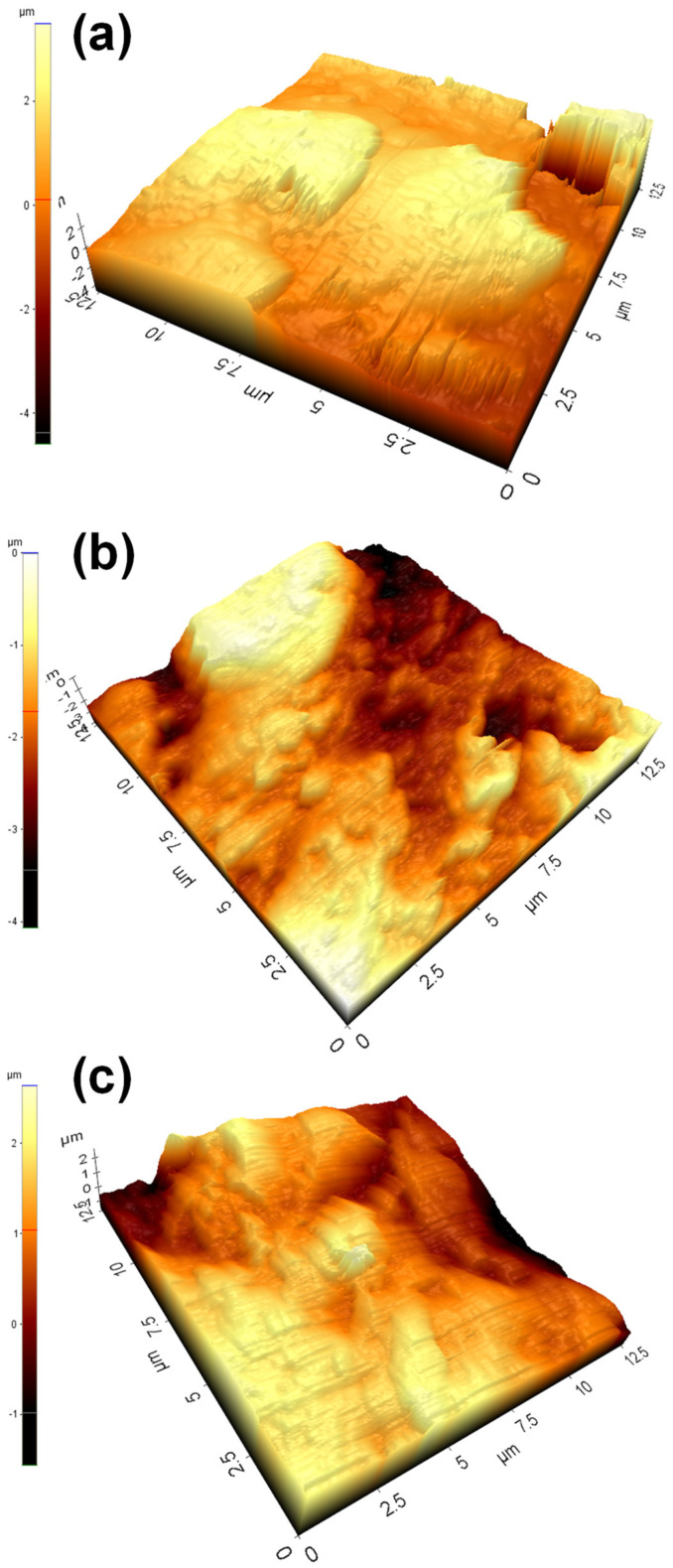
Atomic force microscopic (AFM) micrographs of (**a**) PEEK, (**b**) PEEK/GL13K, and (**c**) PEEK/GL13K-EDC samples.

To further verify the smoothness seen in the micrograph, the surface topography was examined using AFM, as shown in Figure 2. The root mean square roughness (Rq) values of the PEEK, PEEK/GL13K, and PEEK/GL13K-EDC samples were 0.697, 0.634, and 0.530 µm, respectively. The PEEK/GL13K and PEEK/GL13K-EDC samples showed smoother surface topographies than pure PEEK, which further confirmed the successful peptide coating on the PEEK surface.

### 2.2. Surface Properties

**Figure 3:**
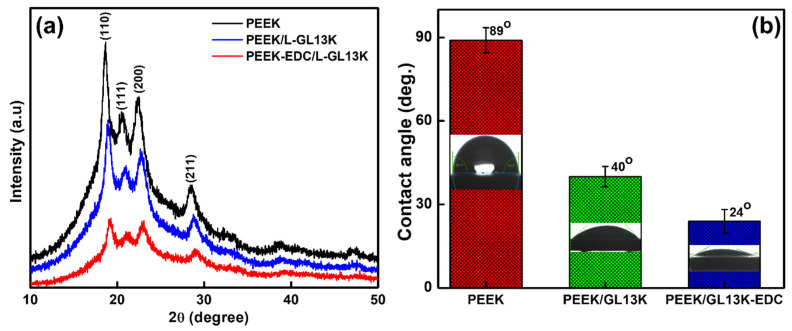
(**a**) X-ray diffraction (XRD) and (**b**) contact angle measurements for PEEK, PEEK/GL13K, and PEEK/GL13K-EDC samples. At least three different spots on each sample surface were measured, and the average values are presented with standard deviation (*n* = 3).

The XRD patterns of the PEEK, PEEK/GL13K and PEEK/GL13K-EDC samples illustrated a semi-crystalline behavior, as represented in Figure 3a. The four diffraction peaks at 2θ of 18.7°, 20.7°, 22.8°, and 28.8° corresponded to the (110), (111), (200) and (211) planes of the PEEK, respectively [37]. The XRD peak intensities of the PEEK/GL13K and PEEK/GL13K-EDC samples were reduced compared with the pure PEEK sample due to the strong peptide coating effects.

The contact angles of the PEEK- and GL13K-coated samples are shown in Figure 3b. The pure PEEK displayed a static water contact angle of 89°, which confirmed its hydrophobic surface. This contact angle result was in line with the data from the literature report (90°) [38]. After GL13K coating, the surface contact angles decreased to 40° and 24° for the PEEK/GL13K and PEEK/GL13K-EDC samples, respectively. The surface change properties further validated the hydrophilicity of the peptide coating on the PEEK surface. Similar trends of decreasing water contact angles after AMP coating on a PEEK sample were reported in the literature [15,17,33]. Moreover, the EDC coupling sample had a higher peptide coating efficiency on the PEEK surface. Therefore, the hydrophilicity of this surface was increased compared with that without EDC treatment. These wettability data further validated our biomimetic peptide coupling strategy on a PEEK substrate.

### 2.3. Chemical Composition Analysis

EDX mapping and the weight percentages of the elements C (85.57%) and O (14.43%) for the pure PEEK sample were examined and matched with the theoretical PEEK stoichiometric chemical composition [39]. The EDX elemental compositions of the PEEK/GL13K and PEEK/GL13K-EDC samples showed lower carbon (84.78% and 83.89%, respectively), lower oxygen (13.62% and 12.60%, respectively), and higher nitrogen (1.62% vs. 2.11%, respectively) contents than the pristine PEEK, as shown in Figure 1. PEEK is a nitrogen-free material [33], and the elemental nitrogen group represents the amide group in the GL13K peptide coating. The richer nitrogen composition in PEEK/GL13K-EDC confirmed that EDC was able to facilitate peptide grafting and achieved 30% more peptide loading than without EDC.

**Figure 4:**
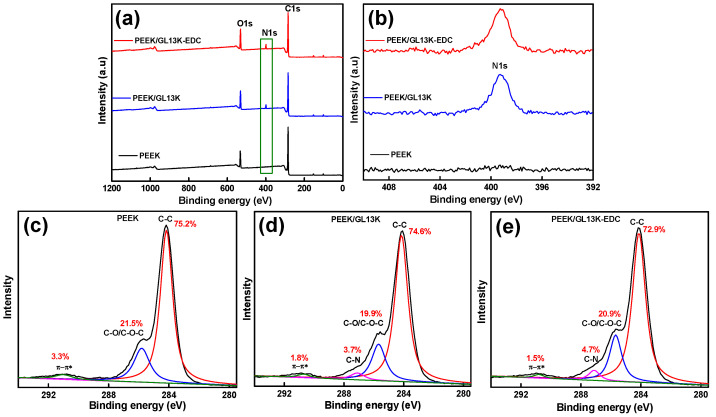
X-ray photoelectron spectroscopy (XPS) spectra of (**a**) full scans, (**b**) detailed scans of N1s, and (**c**–**e**) detailed scans of C 1s in PEEK, PEEK/GL13K, and PEEK/GL13K-EDC samples.

The full XPS spectra of the PEEK, PEEK/GL13K, and PEEK/GL13K-EDC samples are shown in Figure 4a. The pure PEEK sample displayed two distinct XPS peaks at 284.5 eV and 532.3 eV, representing the C 1s (85.5 at%) and O 1s (14.44 at%), respectively. The XPS data closely matched the theoretical PEEK stoichiometric atomic percentages of carbon (86.36 at%) and oxygen (13.64 at%) [39]. The PEEK/GL13K and PEEK/GL13K-EDC samples gave the three distinct XPS signals at 284.7 eV, 532.5 eV, and 398 eV, which represented C 1s (79.36 and 77.49 at%), O 1s (16.63 and 17.27 at%) and N 1s (4.02 and 5.24 at%), respectively. The presence of nitrogen confirmed the GL13K peptide coating on the PEEK surface. Furthermore, the EDC sample showed a 30% higher nitrogen content than the sample without EDC coupling, which further validated the higher peptide coating effect of EDC.

Detailed scans of the N1s spectra of the PEEK, PEEK/GL13K, and PEEK/GL13K-EDC samples are shown in Figure 4b. The PEEK sample did not show an N1s signal due to the absence of nitrogen. The binding energy of the N1s peak at 398.1 eV in the PEEK/GL13K and PEEK/GL13K-EDC samples indicates that the GL13K peptide was successfully coated or immobilized on the surface of the PEEK. There was no obvious peak shift or difference between the PEEK/GL13K and PEEK/GL13K-EDC samples in the detailed scans, but the intensity of the N1s spectra increased in the EDC coupling sample. Both the EDX and XPS results confirmed that EDC coupling increased 30% with GL13K peptide grafting on the PEEK surface.

The deconvolution C 1s peaks of the PEEK, PEEK/GL13K, and PEEK/GL13K-EDC samples are represented in Figure 4c–e. The C 1s spectra of the pristine PEEK surface could relate to the two distinct binding energy peaks at 285.7 eV and 284.3 eV, attributed to the ether bond of C–O–C and the aliphatic group of C–H/C–C bonds in the PEEK’s structure. The small satellite peak at 291.2 eV represents the occurrence of π → π* transitions in electrons in the aromatic ring of the PEEK [40]. There was significant intensity at 286.5 eV, equivalent to the C–N bond in the PEEK/GL13K sample. This is the characteristic signal of the GL13K peptide (Figure 4d,e). C–N binding was higher in the EDC coupling sample (4.7%) than in that without EDC treatment (3.7%). This confirmed the higher GL13K peptide level in the PEEK/GL13K-EDC sample.

### 2.4. Antibacterial Studies

**Figure 5:**
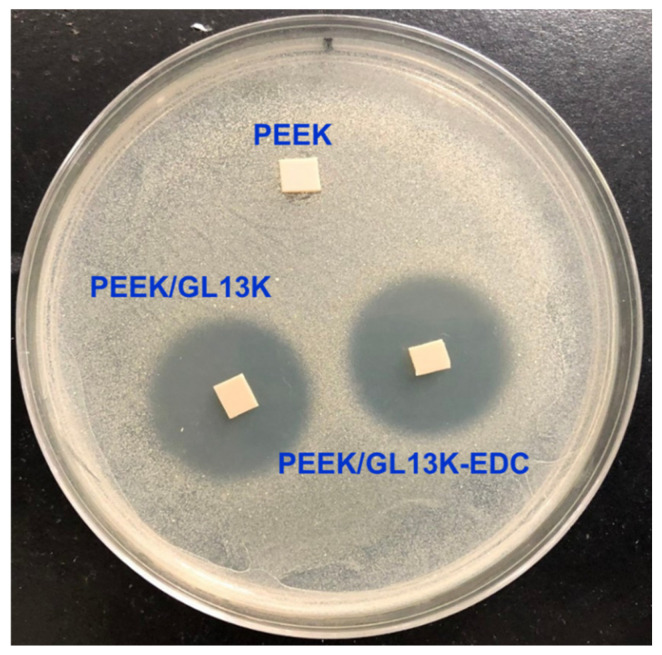
Photographic image and the zone of inhibition against *S. aureus* using PEEK, PEEK/GL13K, and PEEK/GL13K-EDC samples.

The zone of bacterial inhibition and the quantitative analysis of effectivity against *S. aureus* using PEEK, PEEK/GL13K, and PEEK-EDC/GL13K are displayed in Figure 5. The pure PEEK did not demonstrate any bacterial inhibition. The PEEK/GL13K and PEEK/GL13K-EDC samples had inhibition zones of 25 ± 1.35 mm and 28 ± 1.10 mm, respectively. In our previous work on the solution phase, the positive charge of GL13K interacted with the bacteria and caused cell wall collapse and nano- or micrometric pore formation, leading to a conformation structural change in the bacteria [21]. Similarly, the present work indicates cell wall damage and anti-adhesive effects when in contact with the GL13K-coated PEEK sample resulting from the electrostatic interaction, and the highly hydrophilic surface improved bactericidal activity against *S. aureus*.

### 2.5. Post-Bacterial Analysis

**Figure 6:**
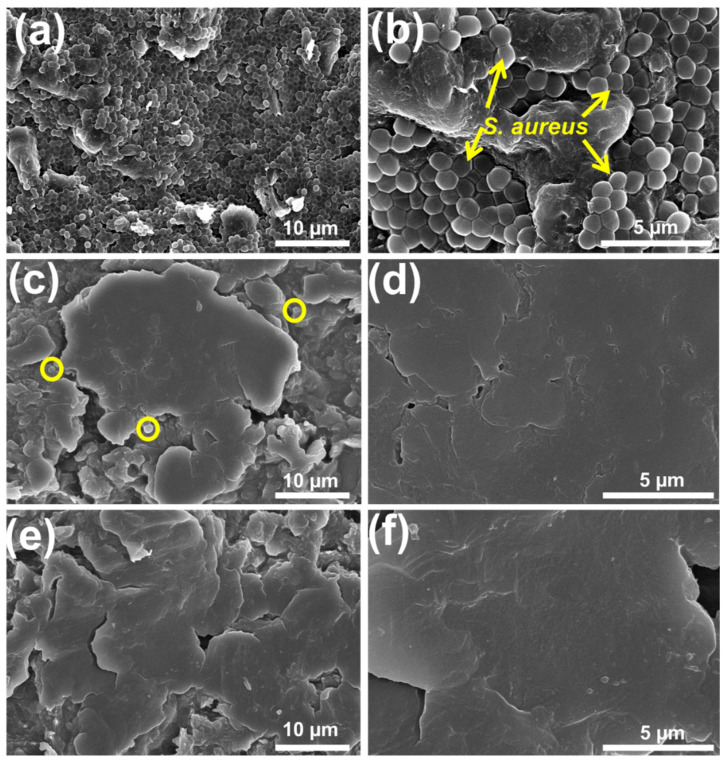
Low- and high-magnification FESEM micrographs of post-bacterial treatment using (**a**,**b**) PEEK, (**c**,**d**) PEEK/GL13K, and (**e**,**f**) PEEK/GL13K-EDC samples.

To further study bacterial attachment, post-bacterial analysis was performed using FESEM (Figure 6a–f). The control PEEK sample had a dense *S. aureus* population, and the bacteria were homogeneously distributed on the surface (Figure 6a,b) due to surface roughness and hydrophobicity [8,38]. In the case of the PEEK/GL13K sample, only very few *S. aureus* bacteria were found (Figure 6c). This might be due to the moderate hydrophilicity and micro-roughness of the PEEK/GL13K surface. Notably, the *S. aureus*-treated PEEK/GL13K-EDC sample showed negligible bacterial adhesion and superior biofilm resistance (Figure 6e) due to its high hydrophilicity and smooth surface. This highly hydrophilic surface could maintain the simultaneous functions of biofilm resistance and soft tissue attachment for implantable applications [7,19]. Thus, the developed coating was effective at inhibiting bacterial attachment. Interestingly, the intact smooth surface of the peptide coating on the surface of the PEEK (with and without EDC grafting) was retained after *S. aureus* treatment, as evident in the higher-magnified FESEM micrographs (Figure 6d,f).

### 2.6. Peptide Coating Stability

**Figure 7:**
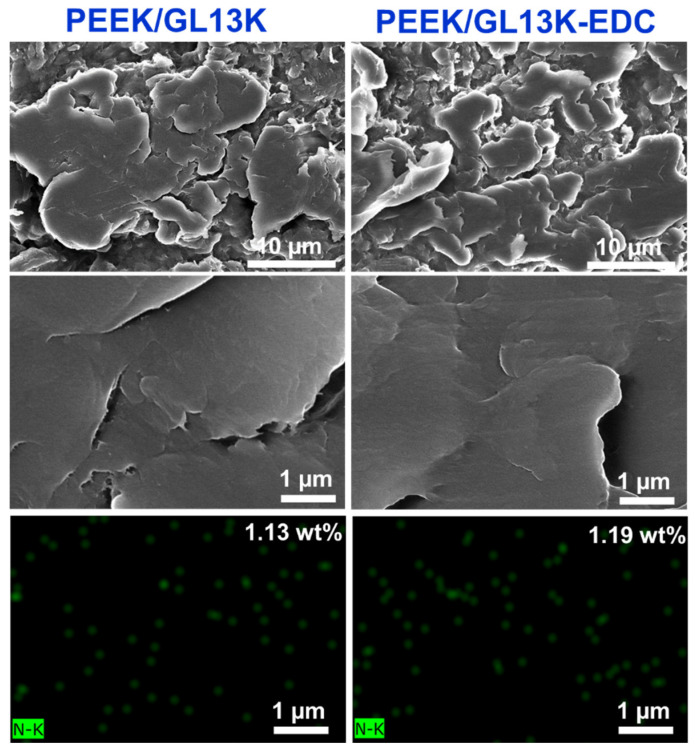
FESEM micrographs (first two rows) and nitrogen mapping derived from EDX mapping (bottom row) of the GL13K released from the PEEK/GL13K and PEEK/GL13K-EDC samples after 24 h of immersion in DI water.

To further investigate the stability of the GL13K coating on the PEEK surface, the coated samples were immersed in DI water for 24 h (which was similar to the bacterial incubation time). The surface morphologies of the PEEK/GL13K and PEEK/GL13K-EDC samples after immersion are displayed in Figure 7. The FESEM morphologies of both peptide-released samples showed comparably smooth surface structures. However, rougher surface regions were observed on the peptide-released samples when compared with the original peptide-coated samples (as displayed in Figure 1). This further confirms that GL13K peptides were partially released from the PEEK surface. Moreover, EDC coupling manifested greater GL13K-releasing behavior than the sample without EDC treatment. The nitrogen contents of both the PEEK/GL13K and PEEK/GL13K-EDC samples (Figure 7) were analyzed using EDX. The original PEEK/GL13K and PEEK/GL13K-EDC samples (as shown in Figure 1) showed nitrogen contents of 1.62 and 2.11 wt%, respectively. These values decreased to 1.13 and 1.19 wt% after water immersion. The PEEK/GL13K-EDC showed a 43.6% (from 2.11 to 1.19 wt% nitrogen content) GL13K release rate, and that of PEEK/GL13K was 30.2% (from 1.62 to 1.13 wt%). Although EDC enhanced GL13K bonding by 30%, the grafted GL13K was able to release in its free form and interact with *S. aureus*. The GL13K concentrations in the DI water after PEEK sample immersion were measured and determined to be equivalent to the released peptide amount. The peptide graft levels were in the range of micrograms per square centimeter. The significant increase in the amount of GL13K peptide released from the EDC-assisted PEEK strongly correlated with the increase in the zone of bacterial inhibition (Figure 5). Furthermore, sufficient coating remained (56.4–69.3% grafted amounts) on the surface to resist biofilm formation (Figure 6d,f).

**Figure 8:**
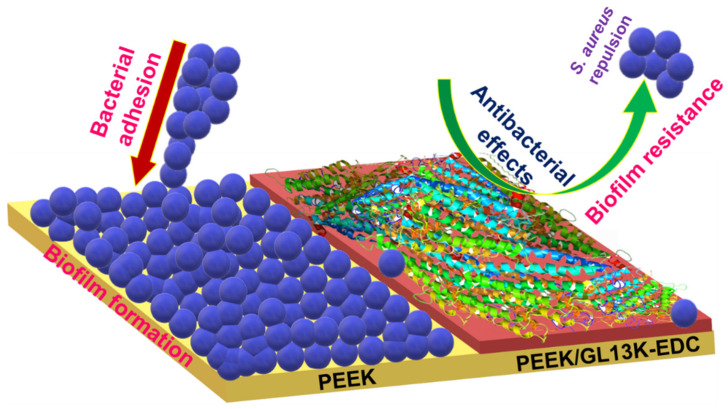
Schematic illustration of bacterial adhesion and the antibacterial activity with biofilm resistance using PEEK and PEEK/GL13K-EDC samples. The red arrow indicates the bacterial adhesion on pure PEEK surface and the green arrow represents antibacterial and biofilm resistance effects on PEEK/GL13K-EDC.

**Figure 9:**
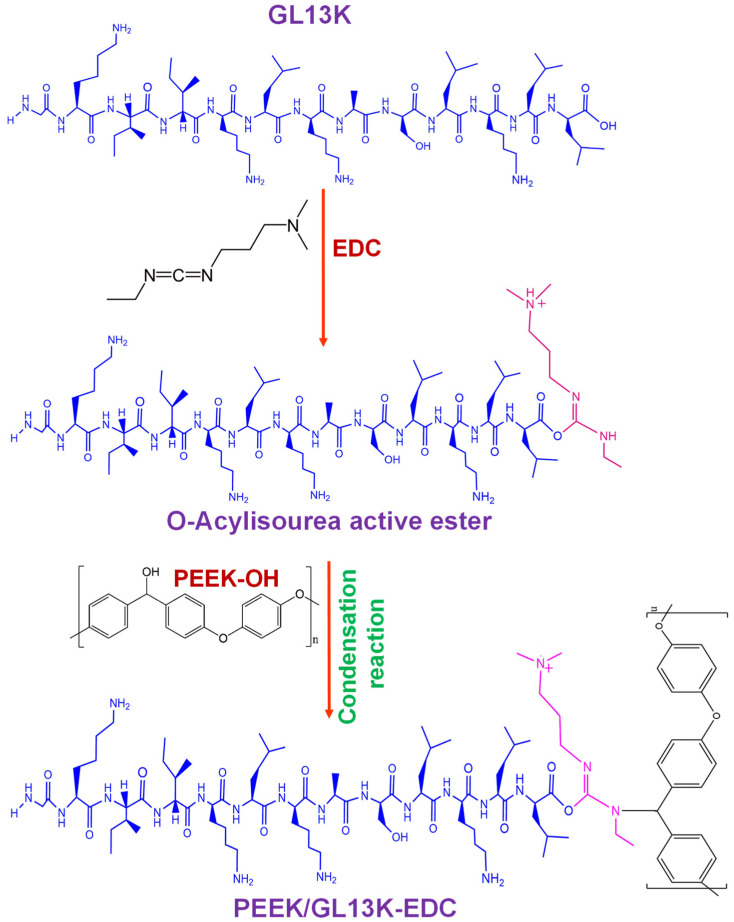
Proposed schematic of the possible chemical attachment of GL13K to the PEEK surface through EDC coupling reactions.

Schematic illustrations of *S. aureus* attachment on pure PEEK and the bacterial anti-adhesion behavior of the GL13K-coated PEEK are displayed in Figure 8. The hydrophobicity and greater surface roughness of pure PEEK can increase *S. aureus* adhesion and induce mature dense biofilm formation [41]. As such, no antibacterial activity against *S. aureus* was observed in the pure PEEK sample. In the case of the PEEK/GL13K-EDC sample, the coating efficiency was improved compared with that without EDC. The zero-length EDC coupling reaction between PEEK and GL13K (Figure 9) helped to release the peptide more effectively when in contact with the biological medium. As such, the amount of peptide released from the PEEK surface was greater in the PEEK/GL13K-EDC sample, and this increased the zone of inhibition (Figure 5). We reported recently that GL13K caused *E. coli* cell wall collapse and induced nano- and micrometer-sized pores. Those led to transmembrane channels and pore formation [21]. *S. aureus* may be disinfected via the same mechanism. The presence of the GL13K peptide on the PEEK surface disturbs or prevents initial *S. aureus* adhesion, thereby restricting bacterial growth and completely reducing biofilm formation by inducing bacterial lysis and anti-adhesion effects [33]. However, the efficiency of using GL13K-coated PEEK for cell attachment and in vivo biomedical studies needs to be further addressed in future work. Moreover, it is important to study the graft level changes over a longer time. This will help investigations to design suitable peptide loadings for specific applications.

## 3. Materials and Methods

### 3.1. Preparation of PEEK/GL13K-EDC

In this work, the PEEK sample (0.5 mm thick, Goodfellow, Huntingdon, UK) was cut into a square shape (5 mm × 5 mm diameter) and directly used for AMP modification. Before peptide coating, the PEEK samples were washed with deionized (DI) water, acetone, ethanol, and DI water again under ultra-sonication for 15 min each, followed by drying under a hot air oven to obtain cleaned PEEK. The required amount of 1-ethyl-3-(3-dimethylaminopropyl)carbodiimide (EDC) (Sigma Aldrich, St. Louis, MO, USA) was dissolved in DI water and mixed with 0.5 µg mL^−1^ of GL13K peptide (from Genomics, New Taipei City, Taiwan). The solution was stirred continuously for 30 min at room temperature. The clean PEEK sample was immersed in the peptide solution and placed in a dark environment for 24 h at ambient temperature. The sample was gently removed from the solution and rinsed with DI water. The sample was dried at room temperature to obtain the PEEK/GL13K-EDC sample. EDC is a zero-length crosslinker that consists of carboxyl and amine-reactive groups [42], and it is favorable for mediating the chemical attachment between antimicrobial peptides (GL13K) and the PEEK polymeric substrate. The proposed schematic of the possible formation of a PEEK/GL13K-EDC sample through EDC coupling reactions [43,44,45] is shown in Figure 9. A similar procedure was followed to prepare a PEEK/GL13K sample without the addition of an EDC coupling agent. The proposed reaction mechanism is shown in Figure S1. The GL13K graft levels were in the range of micrograms per square centimeter, as derived from the mass spectroscopy analysis.

### 3.2. Characterizations of Composite Samples

The surface microstructures of the PEEK, PEEK/GL13K, and PEEK/GL13K-EDC samples were evaluated using a field emission scanning electron microscope (FESEM, SU8220, Hitachi, Tokyo, Japan), and the chemical composition was determined by energy dispersive X-ray spectroscopy (EDX, XF3152, Bruker Taiwan co. Ltd., Zhubei City, Taiwan). An atomic force microscope (AFM, Bruker, Billerica, MA, USA) was used to analyze the surface topography of the samples via the contact mode. X-ray diffraction (XRD, D5005D, Siemens AG, Munich, Germany) was used to examine the crystalline structure. The elemental compositions and chemical structures of the samples were studied using X-ray photoelectron spectroscopy (XPS, VG Microtech MT-500, Thermo Fisher Scientific Inc., Waltham, MA, USA). The hydrophilic/hydrophobic properties of the pure PEEK and peptide-coated PEEK samples were determined via the sessile drop water contact angle (G10-MK2, Kruss GmbH, Hamburg, Germany).

The pristine and coated PEEK samples were immersed in DI water for 24 h, and the morphologies and chemical compositions of these samples were determined. The supernatant water was analyzed after the immersion test for peptide concentration, according to the HPLC-MS/MS procedure described in [46,47], except that a precursor ion quantifier node using dimethyl quantification was employed.

### 3.3. Agar Diffusion Assay for Antibacterial Studies

An agar diffusion assay was used to study the antibacterial activities. In detail, 100 µL of *S. aureus* (BCRC 10781, Bioresource Collection and Research Center, Hsinchu, Taiwan) bacterial suspension was placed on 90-mm petri dishes containing Luria-Bertani (LB) broth and agar medium. Then, the PEEK, PEEK/GL13K, and PEEK/GL13K-EDC samples were placed on the petri dishes containing *S. aureus* and stored in an incubator for 24 h at 37 °C. The bactericidal activities in the zone of inhibition were expressed in millimeters (mm).

## 4. Conclusions

This study illustrates a facile method for the preparation of peptide-grafted PEEK as an antibacterial biomaterial. The direct wet bathing of the GL13K peptide or a GL13K-EDC mixture can be used to successfully coat the peptide onto the PEEK surface, but the EDC coupling samples achieved a better coating efficiency. The chemical compositions, derived from EDX and XPS data, further confirm the 30% greater coating of GL13K onto PEEK/GL13K-EDC than was achieved with PEEK/GL13K. This PEEK/GL13K-EDC sample exhibited a smoother surface, lower surface roughness, and higher hydrophilicity compared with the PEEK/GL13K and PEEK samples. The pristine PEEK did not inhibit bacterial growth and was prone to bacterial colonization. The PEEK/GL13K-EDC sample exerted high antibacterial activity (inhibition zone of 28 mm) and strong biofilm resistance against *S. aureus* bacteria (i.e., no bacterial attachment) compared with the PEEK/GL13K sample (inhibition zone of 25 mm and minor bacterial attachment). These GL13K coatings were stable, and more than half of the grafted peptide was retained on the PEEK after 24 h of immersion in water. In summary, the proposed one-pot protocol was effective at immobilizing the peptide on the polymers. It is a promising approach to modifying implantable medical devices with antibacterial activities and biofilm resistance.

## Data Availability

Not applicable.

## Supplementary Materials

The following supporting information can be downloaded at: https://www.mdpi.com/article/10.3390/ijms23010359/s1.
