# Supplementary material for: Facilitating GL13K Peptide Grafting on Polyetheretherketone via 1-Ethyl-3-(3-dimethylaminopropyl)carbodiimide: Surface Properties and Antibacterial Activity
Source: Int J Mol Sci. 2021 Dec 29;23(1):359. doi: 10.3390/ijms23010359 (PMC8745129; doi:10.3390/ijms23010359)
Supplement: Supplementary file 1 [file ijms-23-00359-s001.zip › ijms-1518592-supplementary.pdf]

Supplementary information

Facilitating GL13K Peptide Grafting on Polyetheretherketone via 1-Ethyl-3-(3-Dimethylaminopropyl)Carbodiimide: Surface Properties and Antibacterial Activity

Chih-Chien Hu [1], Selvaraj Rajesh Kumar [2], Truong Thi Tuong Vi [3], Yu-Tzu Huang [4,5], Dave W. Chen [6] and Shingjiang Jessie Lue [1,2,7,*]

[1] Division of Join Reconstruction, Department of Orthopedics, Chang Gung Medical Center at Linkou, Guishan District, Taoyuan City 333, Taiwan; chihchienhu@hotmail.com
[2] Department of Chemical and Materials Engineering, Chang Gung University, Guishan District, Taoyuan City 333, Taiwan; rajeshkumarnst@gmail.com
[3] Division of Pediatric Gastroenterology and Hepatology, Department of Pediatrics, Chang Gung Memorial Hospital, Guishan District, Taoyuan City 333, Taiwan; truongthituongvi005@gmail.com
[4] Department of Chemical Engineering, Chung Yuan Christian University, Zhongli, Taoyuan City 320, Taiwan; yt_huang@cycu.edu.tw
[5] R&D Center for Membrane Technology and Research Center for Circular Economy, Chung Yuan Christian University, Zhongli, Taoyuan City 320, Taiwan
[6] Department of Orthopedic Surgery, Chang Gung Memorial Hospital, Keelung City 204, Taiwan; mr5181@cgmh.org.tw
[7] Department of Safety, Health and Environment Engineering, Ming Chi University of Technology, Taishan District, New Taipei City 243, Taiwan
* Correspondence: jessie@mail.cgu.edu.tw; Tel.: +88-63-2118800 (ext. 5489); Fax: +88-63-2118700

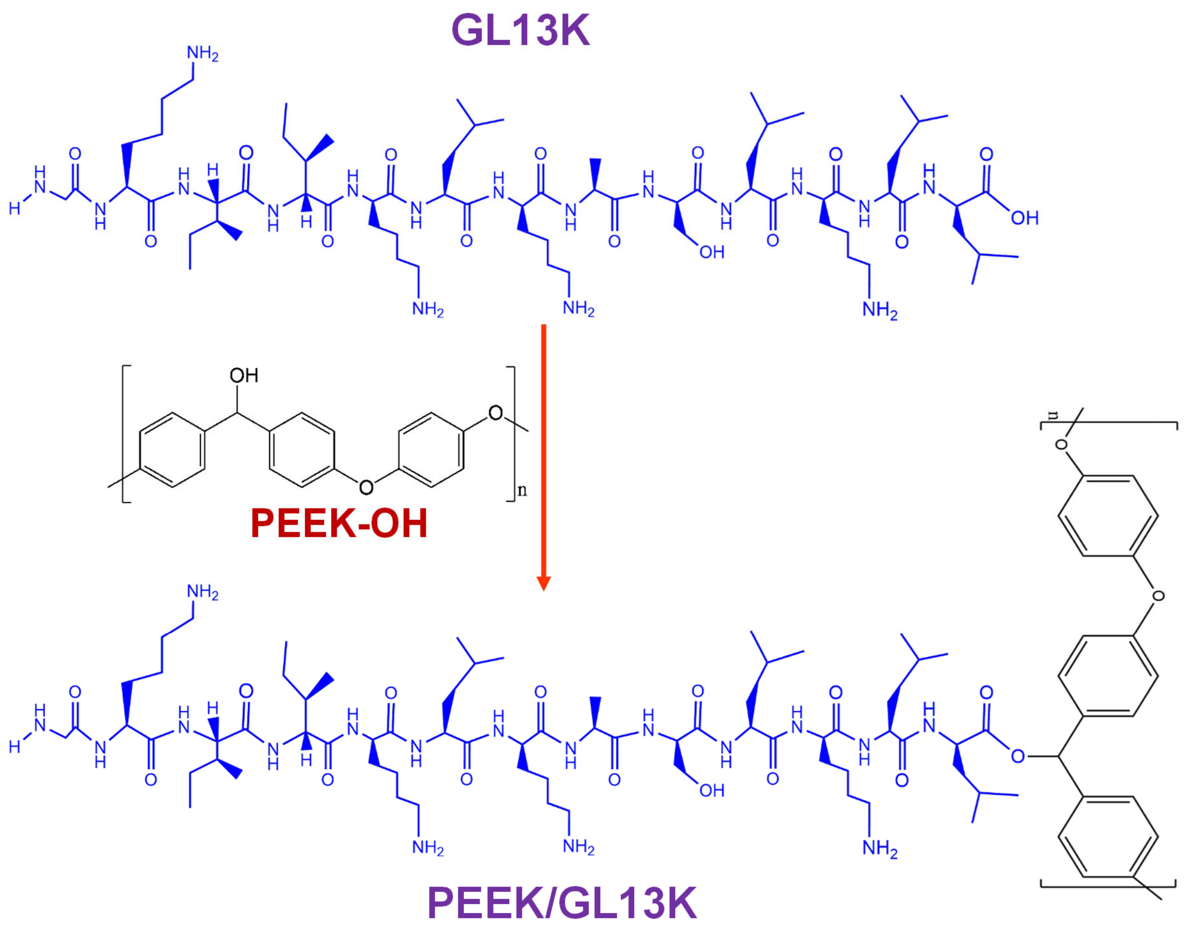

Figure S1. The schematic diagram of the possible GL13K grafting on PEEK surface.
